# Medium chain length polyhydroxyalkanoates consisting primarily of unsaturated 3-hydroxy-5-*cis*-dodecanoate synthesized by newly isolated bacteria using crude glycerol

**DOI:** 10.1186/s12934-016-0454-2

**Published:** 2016-03-17

**Authors:** Amtiga Muangwong, Thanawat Boontip, Jittakan Pachimsawat, Suchada Chanprateep Napathorn

**Affiliations:** Department of Microbiology, Faculty of Science, Chulalongkorn University, Phayathai Road, Patumwan, Bangkok, 10330 Thailand; Program in Biotechnology, Faculty of Science, Chulalongkorn University, Phayathai Road, Patumwan, Bangkok, 10330 Thailand

**Keywords:** Medium chain length PHAs, Crude glycerol, Used cooking oil, *Acinetobacter* sp., *Pseudomonas* sp., *Enterobacter* sp., *Bacillus* sp., 3-Hydroxyoctanoate, 3-Hydroxy-5-*cis*-dodecanoate (3H5DD)

## Abstract

**Background:**

Our study aimed to search for novel bacteria capable of producing polyhydroxyalkanoates (PHAs) using crude glycerol residue obtained from biodiesel production in which used cooking oils were the substrates.

**Results:**

Newly isolated bacteria from soils in Thailand were screened for the efficient production of PHAs from crude glycerol. The bacterial strains were cultivated on glucose, refined glycerol, crude glycerol, or various cooking oils (canola oil, palm oil, soybean oil, sunflower oil, corn oil, grape seed oil, olive oil, rice bran oil, camellia seed oil) for growth and PHA production. The effects of the total organic carbon (TOC) concentration and the mole ratio of carbon to nitrogen were investigated in batch cultivation. ^1^H NMR, two dimensional-^1^H-correlation spectroscopy (2D-^1^H-COSY) and ^13^C NMR analyses confirmed four bacterial strains were capable of producing medium-chain-length PHAs (mcl-PHAs), consisting of 3-hydroxyoctanoate (3HO) and 3-hydroxy-5-*cis*-dodecanoate (3H5DD), from crude glycerol. On the basis of phenotypic features and genotypic investigations, the bacterial strains were assigned as: ASC1, *Acinetobacter* genus (94.9 % similarity); ASC2, *Pseudomonas* genus (99.2 % similarity); ASC3, *Enterobacter* genus (99.2 % similarity); ASC4, *Bacillus* genus (98.4 % similarity). The highest amount of mcl-PHAs, 17.5 ± 0.8 g/L (content 61.8 ± 3.3 % wt), with 3HO (14.7 ± 2.2 mol %), 3H5DD (85.3 ± 2.2 mol %), and a total biomass of 32.3 ± 0.3 g/L, was obtained from *Pseudomonas* sp. ASC2 in batch cultivation after 36 h. The mcl-PHAs recovered had a number-average molecular weight (*M*_N_) of 3.6 × 10^4^ Da. Homopolymeric 3H5DD was obtained when the cultivation time was prolonged to 96 h.

**Conclusions:**

Novel PHA-producing strains were isolated and identified. These bacterial strains are able to produce mcl-PHAs from crude glycerol. The mcl-PHAs produced contained a high percentage of 3H5DD, which suggests their future application as softeners mixed with other biomaterials. The unsaturated side chain of 3H5DD monomers containing double bounds offers additional potential for improving the properties of the mcl-PHAs or extending their applications to the food industry.

## Background

Policy changes in various countries to enforce the use of biomass-derived fuels and bio-based polymers are current trends worldwide due to the depletion of fossil resources within the coming decades as well as global warming issues [1]. The sustainability of these bio-based industries has become an important concern that calls for a business strategy that integrates social, safety, health and environmental benefits with the technological and economic objectives of their activities [2]. The current trend to change the feedstock used from hydrocarbons to biological compounds has radically altered the technological basis of the biotechnology industry. Primarily, there is a need for technologies that enable the economical processing of complex biomass; for example, the production of biodiesel from renewable resources and the incorporation of the production of biodegradable polymers from organic wastes obtained from biodiesel processes remains a major challenge requiring innovation and technological developments.

Polyhydroxyalkanoates (PHAs) are a family of biodegradable polyesters synthesized by various types of microorganisms. Taking advantage of the diversity of microorganisms, PHAs can be produced from a variety of materials depending on the ability of the PHA producing strains. A good starting point is to link the production of biodegradable PHAs with the rapidly emerging bioenergy industries, including the use of organic waste from biodiesel production. This strategy takes the principle of life cycle assessment into consideration. The proposed use of waste residue is advantageous from the viewpoint of the life cycle of the microorganism used because it removes the direct effect of PHA production on land usage for agriculture, raw materials such as human food and animal feeds, CO_2_ emissions from agricultural activities and the use of chemicals for farming [3–5]. However, there is a big gap between economic drivers and societal perceptions that will promote or hinder advancements in the use of wastes as resources for a bio-based society.

Crude glycerol is an organic waste from biodiesel production. The increasing amount of biodiesel production has generated crude glycerol residues with a 98 % conversion yield, resulting in 1 kg of crude glycerol produced for every 10 kg of biodiesel manufactured [6]. The dramatic rise in the world’s biodiesel production has resulted in a large surplus of crude glycerol production and its limited market size has resulted in a decrease in its price. Crude glycerol is of low-impact economic value because of the presence of various impurities such as methanol, soap, free fatty acids, fatty acid methyl esters, and spent catalyst residues. Nowadays, there are a growing number of attempts to develop chemical processes and bioprocesses for the value-added conversion of crude glycerol [7–9]. The development of a bioconversion system to convert crude glycerol residue to higher value-added products is urgently needed and an opportunity to overcome the negative impact of low prices of crude glycerol. Thus, microbial fermentation to convert crude glycerol to useful chemicals such as PHAs is an attractive solution. In general, almost all biodiesel is produced from vegetable oils. In Brazil, a world leader in biofuels, a range of oils, including soybean, sunflower, castor oil, and *Jatropha curcas* are used, while in Europe the majority of biodiesel is produced from rapeseed oil. However, used cooking oils are becoming increasingly popular as starting materials because of their lower cost, because they are not a human food resource, and to reduce problems in municipal waste management, etc. [10]. The chemical composition of crude glycerol is highly variable with different types of catalysts and starting materials used for biodiesel production [9]. Hansen et al. (2009) analyzed the chemical composition of 11 types of crude glycerol obtained from seven Australian biodiesel manufacturers and revealed that the glycerol content in crude glycerol varied from 38–96 %, and up to 16.1 % methanol was present as an impurity [11]. Moreover, Hu et al. (2012) reported that crude glycerol obtained during biodiesel preparation from waste vegetable oil contained glycerol 28.9 (wt %), an organic fraction 52.3 (wt %), and 8.6 % methanol. The aqueous fraction consisted mainly of glycerol, methanol and water, and the organic fraction contained fatty acid methyl esters, free fatty acids, and glycerides [12].

The objective of this work was to search for new bacteria capable of utilizing crude glycerol obtained from the biodiesel industry in Thailand, where the starting material for the biodiesel reaction is used-cooking oils, for growth and PHA production. Our efforts in screening new bacterial strains capable of using the crude glycerol directly for PHA production are reported. We discuss the biosynthesis and characterization of medium chain length (mcl)-PHAs and the context for PHA production.

## Results and discussion

### Morphological and taxonomical identification

A total of 20 isolates were obtained from soil that was contaminated with used-cooking oil using enrichment methods, and these isolates were able to grow on medium containing crude glycerol from the biodiesel industry. Using the methods described in “Methods” section for screening PHA producing bacteria, four different strains showed positive results, defined as exhibiting the accumulation of poly-3-hydroxybutyrate (PHB) granules; these strains were named ASC1, ASC2, ASC3 and ASC4, respectively. The physiological and biochemical characteristics of these strains were examined (Table 1). Colonies of ASC1 on nutrient agar plates were small, circular, slightly convex with entire margins, opaque and moist. The bacterium ASC1 was Gram-negative with a short rod shape, non-spore forming and non-motile. Colonies of ASC2 on nutrient agar plates were circular with entire margins, light-yellow and shiny. The bacterium ASC2 was Gram-negative, rod-shaped, motile and non-spore forming. Colonies of ASC3 were medium sized, circular with entire margins, opaque and moist. The bacterium ASC3 was Gram-negative, rod-shaped, motile and non-spore forming. Colonies of ASC4 on nutrient agar plates were irregular, opaque and large with undulated margins. The bacterium ASC4 was Gram-positive, rod-shaped, motile and spore-forming. All four strains grew aerobically and were capable of growth at 30 and 37 °C. They were positive in tests for catalase, citrate utilization and ornithine decarboxylase. Only strain ASC3 was able to grow on MacConkey medium, where it reduced lactose and exhibited pink colonies. Strain ASC4 gave a positive result for lysine decarboxylase but could not reduce nitrate.

**Table 1:** Characterization of isolated PHA producing strains by biochemical tests

Biochemical test	Results			
	ASC1	ASC2	ASC3	ASC4
Motility	−	+	+	+
Oxidase	−	+	−	+
Catalase	+	+	+	+
Indole production	−	−	−	−
Methyl red	−	−	−	−
Voges-Proskauer	−	−	+	−
Citrate (simmons)	+	+	+	+
Triple sugar iron (TSI) reaction	K/K	K/K	A/A	K/A
H_2_S production (TSI)	−	−	−	−
Lysine decarboxylase	−	−	−	+
Ornithine decarboxylase	+	+	+	+
Urease	−	−	+	+
Nitrate reduction	+	+	+	−

*K/K* alkaline slant/alkaline butt = peptone was used and no carbohydrates were fermented, *A/A* acid slant/acid butt = glucose, lactose and sucrose were fermented, *K/A* alkaline slant/acid butt = only glucose was fermented and peptone was used

**Fig. 1:**
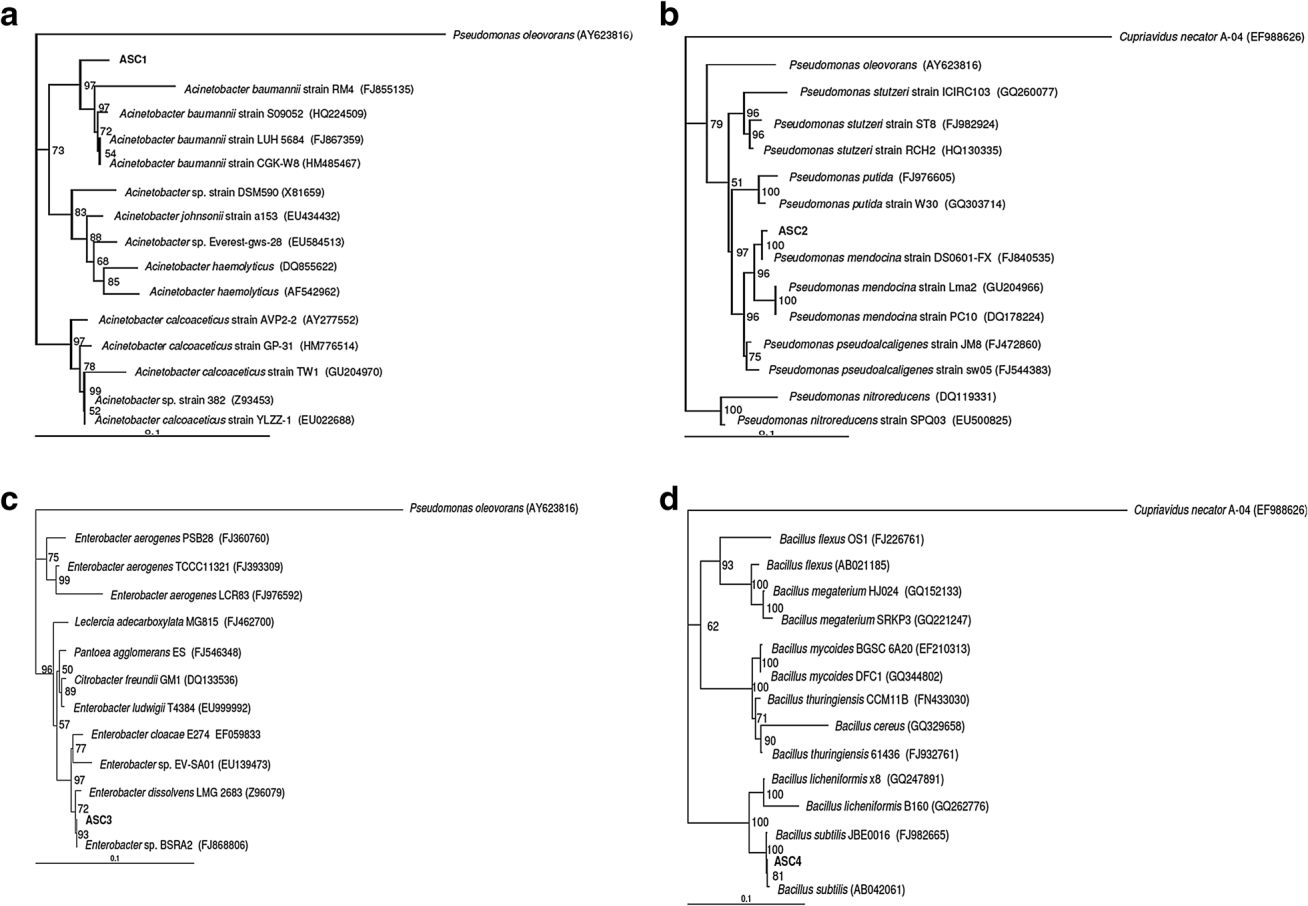
Phylogenetic tree constructed based on 16S rDNA sequences. **a** *Acinetobacter* sp. ASC1. **b** *Pseudomonas* sp. ASC2. **c** *Enterobacter* sp. ASC3. **d** *Bacillus* sp. ASC4. The trees were constructed by the neighbor-joining method and rooted by referring to *Pseudomonas oleovorans* or *Cupriavidus necator* A-04. The numbers at branches refer to the percentage confidence estimated by a bootstrap analysis with 100 replications. The analysis was performed by including *16S rDNA* gene sequences from GenBank (accession numbers indicated in parentheses). *Bar* = 0.1 estimated substitutions per sequence position

Physiological and biochemical analysis alone is not sufficient for the accurate characterization of bacteria. To identify these strains, their *16S rDNA* genes were amplified by PCR using genomic DNA as the template. The partial sequences of the *16S rDNA* genes of the strains ASC1 (1536 bp), ASC2 (1465 bp), ASC3 (1453 bp) and ASC4 (1503 bp) were submitted to the GenBank sequence database [GenBank: GU227612, GU227613, GU227614, GU227615]. According to a phylogenic analysis, the nucleotide sequence of the DNA fragment encoding the *16S rDNA* gene of strain ASC1 clearly demonstrated that the closet matches belonged to the genus *Acinetobacter*, with the highest identity (94.9 %) to *A. baumannii* strain RM4 [GenBank: FJ855135]. Figure 1a shows a phylogenetic tree generated by the neighbor-joining method. The neighbor-joining analysis of the *16S rDNA* gene was rooted by referring to a gammaproteobacterium (*Pseudomonas oleovorans*) and comparing among *Acinetobacter* spp. with a bootstrap support value of 100 %. The strain ASC1 was identified as *Acinetobacter* sp. and named *Acinetobacter* sp. ASC1. Strain ASC2 belonged to the genus *Pseudomonas*, with the highest identity (99.2 %) to *P. mendocina* strain DS0601-FX [GenBank: FJ840535]. A phylogenetic tree generated by the neighbor-joining method is shown in Fig. 1b. Strain ASC2 was identified as *Pseudomonas* sp. and named *Pseudomonas* sp. ASC2. Strain ASC3 belonged to the genus *Enterobacter*, with the highest identity (99.2 %) to *Enterobacter* sp. strain BSRA2 [GenBank: FJ868806] (Fig. 1c). Strain ASC3 was identified as *Enterobacter* sp. and named *Enterobacter* sp. ASC3. Strain ASC4 belonged to the genus *Bacillus*, with the highest identity (98.4 %) to *B. subtilis* strain IAM 12118T [GenBank: AB042061]. See Fig. 1d for a phylogenetic tree. Strain ASC4 was identified as *Bacillus* sp. and named *Bacillus* sp. ASC4.

There have been an increasing number of reports in recent years regarding the use of glycerol as a substrate for PHA production. Most researchers have reported the use of pure glycerol for PHA production, while others have reported on microbial strains that utilize crude glycerol from the biodiesel industry without purification such as the removal of methanol or other impurities. Ashby et al. (2004) reported that *P. oleovorans* NRRL B-14682 and *P. corrugata* 388 were able to produce PHA (total cell dry mass of 1.3 g/L with 13–27 wt % PHA content and 2.1 g/L with 42 % PHA, respectively) from crude glycerol biodiesel byproduct containing glycerol, free fatty acids and fatty acid methyl esters in shaken-flask experiments [13]. Ibrahim and Steinbüchel (2009) achieved a high cell-density and PHA productivity (81.2 g/L cell dry mass with 66.9 wt % PHB content) using *Zobellella denitrificans* MW1 [14]. Cavalheiro et al. (2009) reported on PHB production from crude glycerol by *Cupriavidus necator* DSM 545. In that study, a maximum cell dry mass of 82.5 g/L was obtained using pure glycerol with a 38 wt % PHB content (31.4 g/L). A lower cell dry mass of 68.8 g/L was observed with crude glycerol (14.7 g/L) but it had the same percentage PHB content [15]. Kawata and Aiba (2010) obtained cell dry mass of 4.1 g/L with a 19 wt % PHB content in batch cultures *Halomonas* sp. KM-1 using 3 % crude glycerol [16]. Ibrahim and Steinbuchel (2010) isolated and identified a new PHA accumulating strain of the genus *Zobellella* which was able to use glycerol for growth [14]. Recently, Pappalardo et al. (2014) reported that *P. mediterranea* 9.1 could produce mcl-PHAs from partially refined glycerol, but it only reached a cell dry mass of 3 g/L with 21 wt % PHA content after 72 h [17]. Thus, the abilities of our isolated strains in growth and biosynthesis of PHAs from glucose, refined glycerol, crude glycerol and various cooking oils were tested in subsequent experiments.

### The ability of isolated bacterial strains in growth and biosynthesis of PHAs using glucose, refined glycerol, crude glycerol or various cooking oils

**Fig. 2:**
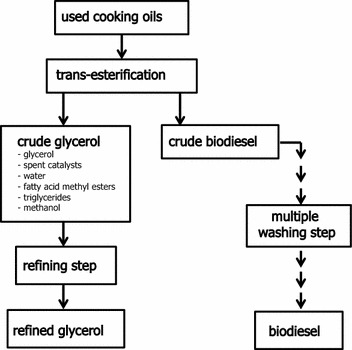
Schematic of the biodiesel production process in which used cooking oils are the substrate

For this study, crude glycerol and refined glycerol were obtained from a biodiesel plant that uses cooking oils as raw material. Figure 2 shows a brief scheme of the biodiesel production from used cooking oils. The composition of refined glycerol was determined by Witcorp Products Ltd. (Thailand). The crude glycerol was subjected to analysis of chemical composition, including glycerol, methanol and free fatty acid content, by HPLC and GC analyses. Summaries of the chemical compositions of the refined glycerol and crude glycerol used in this study are shown in Table 2. It was clear that the refined glycerol, and also the crude glycerol, contained heavy metals and arsenic that limit their pharmaceutical and food applications. Therefore, to solve the waste management issues, we aimed to use microbial conversion to convert the crude glycerol to value-added chemicals without a requirement for refining steps.

**Table 2:** Compositions of refined glycerol and crude glycerol

Analysis	Result	Analytical methods
Refined glycerol		
Glycerol content	99.5 wt % Min	A.O.C.S. Ea 7–50
Specific gravity	1.26 Min	A.O.C.S. Ea 7–50
Color (APHA)	10 Max	Lovibond
Heavy metal	5 ppm Max	BS 5711: Part 15
Water content	0.5 wt % Max	Karl fisher
Combustibles	0.01 wt % Max	USP-30
Arsenic	1.5 ppm Max	BS 5711: Part 10
Diethylene glycol	0.1 wt % Max	USP-30
Sulfate	0.002 wt % Max	USP-30
Iron	10 ppm Max	USP-31
Crude glycerol		
Total organic carbon	300.9 g/L	Total organic carbon analyzer
Glycerol content	50.2 wt %	HPLC
Methanol content	12.0 wt %	GC
Organic fraction	38.4 wt %	GC
Lauric acid (C_12:0_)	1.0 wt %	GC
Palmitic acid (C_16:0_)	5.4 wt %	GC
Stearic acid (C_18:0_)	0.6 wt %	GC
Oleic acid (C_18:1_)	11.9 wt %	GC
Linoleic acid (C_18:2_)	17.7 wt %	GC
Linolenic acid (C_18:3_)	1.8 wt %	GC

Microbial growth and PHA accumulation studies were preliminarily carried out using glucose, refined glycerol, crude glycerol and various cooking oils: canola oil, palm oil, soybean oil, sunflower oil, corn oil, grape seed oil, olive oil, rice bran oil, and camellia seed oil. First, cells were cultured in production medium with 20 g/L carbon source and a carbon to nitrogen (C/N) ratio of 200 for 72 h at 30 °C on a rotary shaker. Table 3 shows the results. All of the bacterial strains could grow on glucose, but only small quantity of biomass and PHA accumulation was observed; the type of PHA produced was PHB. Next, refined glycerol was examined as the carbon source for growth and PHA production. The highest total cell dry mass, 10.0 ± 0.9 g/L, was obtained for *Pseudomonas* sp. ASC2; however, only 3.9 ± 0.2 wt % of PHA was obtained. The composition of PHA produced from refined glycerol consisted of 93 mol % 3-hydroxyoctanoate (3HO) and 7 mol % 3-hydroxy-5-*cis*-dedecanoate (3H5DD) [P(93 % 3HO-co-7 % 3H5DD)]. For comparison with refined glycerol, crude glycerol was used as the carbon source in the same cultivation conditions. A similar cell dry mass was obtained as on use of refined glycerol, but higher PHA accumulation was observed. The crude glycerol consisted of glycerol 50.2 wt %, methanol 12.0 wt %, lauric acid (C_12:0_) 1.0 wt %, palmitic acid (C_16:0_) 5.4 wt %, stearic acid (C_18:0_) 1.6 wt %, oleic acid (C_18:1_) 11.9 wt %, linoleic acid (C_18:2_) 17.7 wt %, linolenic acid (C_18:3_) 1.8 wt %, and residual cooking oils. Crude Glycerol and the fatty acids listed above were used as precursors for PHA production. The various cooking oils were tested for growth and PHA accumulation. Palm oil concentration of 40 g/L gave the highest cell dry mass and PHA content. Canola oil and rice bran oil also promoted cell growth, but less PHA accumulation. It has been reported that the fatty acid composition in palm oil consists of lauric acid (C_12:0_) 0.1 wt %, palmitic acid (C_16:0_) 45 wt %, oleic acid (C_18:1_) 40.5 wt % and linoleic acid (C_18:2_) 10.1 wt % [18, 19]. Thus, from crude glycerol, cells used glycerol and several fatty acids for cell growth, and medium chain fatty acids as the precursors for PHA production.

**Table 3:** PHA production by isolated strains grown on glucose, refined glycerol, crude glycerol or cooking oils

Substrate	Acinetobacter sp. ASC1		*Pseudomonas* sp. ASC2		Enterobacter sp. ASC3		Bacillus sp. ASC4	
	CDM (g/L)	PHAs (wt %)	CDM (g/L)	PHAs (wt %)	CDM (g/L)	PHAs (wt %)	CDM (g/L)	PHAs (wt %)
Glucose	2.7 ± 0.1	8.2 ± 3.5	2.2 ± 0.1	21.7 ± 2.6	2.6 ± 0.2	16.8 ± 1.9	4.8 ± 0.4	18.9 ± 1.5
Refined glycerol	8.6 ± 0.1	3.7 ± 2.4	10.0 ± 0.2	3.9 ± 0.9	7.2 ± 0.1	14.1 ± 2.7	6.1 ± 0.2	6.7 ± 2.8
Crude glycerol	8.5 ± 0.2	25.4 ± 3.1	10.7 ± 0.1	28.2 ± 1.2	9.8 ± 0.3	33.7 ± 1.3	7.8 ± 0.1	34.4 ± 3.4
Canola oil	9.0 ± 0.1	2.2 ± 3.2	12.1 ± 0.2	2.4 ± 2.0	9.2 ± 0.1	1.8 ± 1.4	9.5 ± 0.2	2.5 ± 1.1
Palm oil	13.3 ± 0.4	14.2 ± 3.5	17.8 ± 0.3	5.5 ± 1.2	12.1 ± 0.1	12.0 ± 2.2	14.6 ± 0.3	12.1 ± 2.0
Soybean oil	5.8 ± 0.3	6.7 ± 2.0	9.7 ± 0.1	5.6 ± 2.2	8.1 ± 0.2	5.3 ± 1.8	6.6 ± 0.1	3.4 ± 1.3
Sunflower oil	8.4 ± 0.1	7.3 ± 2.1	7.2 ± 0.1	2.5 ± 1.8	6.5 ± 0.3	5.5 ± 2.0	7.9 ± 0.1	4.7 ± 0.9
Corn oil	5.9 ± 0.2	4.1 ± 1.7	5.1 ± 0.1	2.6 ± 1.5	5.2 ± 0.1	3.1 ± 1.4	5.8 ± 0.3	2.2 ± 1.0
Grape seed oil	5.3 ± 0.1	7.4 ± 1.9	10.2 ± 0.2	5.6 ± 2.5	6.1 ± 0.1	6.3 ± 1.0	6.2 ± 0.1	5.1 ± 1.3
Olive oil	5.3 ± 0.1	5.0 ± 1.0	5.3 ± 0.1	3.7 ± 0.8	4.3 ± 0.2	2.1 ± 0.8	5.0 ± 0.1	3.2 ± 0.7
Rice bran oil	9.9 ± 0.1	5.1 ± 1.2	17.0 ± 0.2	5.9 ± 1.9	10.1 ± 0.1	4.8 ± 1.1	12.5 ± 0.1	6.0 ± 1.5
Camellia seed oil	5.7 ± 0.1	3.4 ± 1.1	5.5 ± 0.1	3.3 ± 1.1	4.8 ± 0.1	2.9 ± 0.6	3.7 ± 0.1	2.2 ± 0.2

Bacteria were cultured in production medium with 20 g/L carbon source at a carbon to nitrogen ratio of 200 for 72 h at 30 °C on a rotary shaker. The dry cell mass was assayed by GC analysis. All data are expressed as ± SD and represent the mean value of three parallel experiments

*CDM* cell dry mass (g/L), *PHA* total PHA content (wt %)

### Effect of crude glycerol concentration and the ratio of carbon to nitrogen

**Fig. 3:**
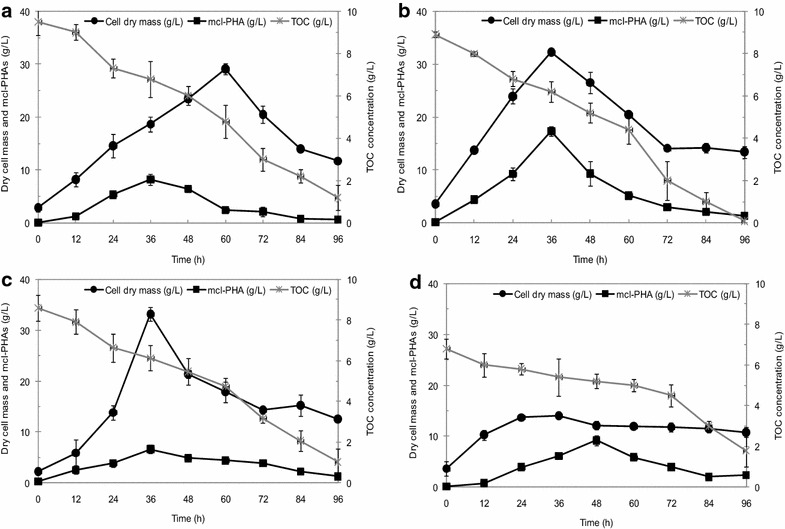
Time courses of cell dry mass (g/L), mcl-PHA levels (g/L) and total organic carbon (TOC) concentration (g/L) in cultures. **a** *Acinetobacter* sp. ASC1. **b** *Pseudomonas* sp. ASC2. **c** *Enterobacter* sp. ASC3. **d** *Bacillus* sp. ASC4. The cells were grown on crude glycerol with a TOC concentration of 10 g/L and a C/N ratio of 200 in a bioreactor in batch mode. Cultures were performed in triplicate. The *error bars* represent standard deviations

Next, bioreactor cultivation was performed in batch mode for *Acinetobacter* sp. ASC1, *Pseudomonas* sp. ASC2, *Enterobacter* sp. ASC3 and *Bacillus* sp. ASC4, respectively, to investigate in detail the accumulation of PHAs using crude glycerol as the carbon source. The concentration of crude glycerol was defined as the total organic carbon concentration (TOC). Initial TOC concentrations varied between 5 and 20 g/L, and C/N was set at 200. The total cell dry mass (CDM) (g/L), residual-cell mass (RCM) (calculated by subtracting the amount of PHA from the total cell dry mass; g/L), PHA content (%), specific growth rate [μ, (1/h)], specific production rate [ρ, (g PHA/g CDM/h)], yield coefficient of PHA produced/g residual-cell mass [Y_P/X_, (g PHA/g RCM)] and productivity (g PHA/(L·h)) were determined (Table 4). The C/N ratio of 200 represents nitrogen deficient conditions. Figure 3 shows time courses of cell dry mass, mcl-PHAs and TOC concentration on growing *Acinetobacter* sp. ASC1 (Fig. 3a), *Pseudomonas* sp. ASC2 (Fig. 3b), *Enterobacter* sp. ASC3 (Fig. 3c) and *Bacillus* sp. ASC4 (Fig. 3d) when grown on crude glycerol at the optimal initial TOC concentration of 10 g/L. *Pseudomonas* sp. ASC2 (Fig. 3b) and *Enterobacter* sp. ASC3 (Fig. 3c) started to accumulate mcl-PHAs during the exponential growth phase. The TOC concentration decreased simultaneously with cultivation time. The nitrogen source, ammonium sulfate, was consumed completely after 12 h of cultivation (data not shown). *Pseudomonas* sp. ASC2 showed the highest PHA productivity attained in the batch cultures (0.6 g PHA/(L·h)) and the highest total cell dry mass (32.3 ± 0.3 g/L), PHA concentration (20.0 ± 0.9 g/L) and PHA content (61.8 ± 3.3 wt %). *Enterobacter* sp. ASC3 yielded a similar total cell dry mass (33.1 ± 0.3 g/L) but a lower PHA concentration (15.6 ± 0.8 g/L) and PHA content (47.2 ± 2.2 wt %).

**Table 4:** Kinetic study of the effect of total organic carbon (TOC) concentration

TOC (g/L)	CDM	PHA	Yield	μ	ρ	Productivity
	(g/L)	Content (wt %)	(g PHA/g RCM)	(1/h)	(g PHA/g CDM/h)	[g PHA/(L h)]
TOC 5 g/L						
*Acinetobacter* sp. ASC1	12.0 ± 0.1	15.6 ± 1.7	0.19	0.033	0.007	0.05
*Pseudomonas* sp. ASC2	12.2 ± 0.2	22.4 ± 2.4	0.29	0.031	0.012	0.08
*Enterobacter* sp. ASC3	13.0 ± 0.2	18.5 ± 3.2	0.23	0.035	0.009	0.07
*Bacillus* sp. ASC4	8.7 ± 0.3	33.5 ± 1.2	0.5	0.018	0.018	0.08
TOC 10 g/L						
*Acinetobacter* sp. ASC1	23.5 ± 0.1	54.6 ± 3.4	0.49	0.023	0.014	0.36
*Pseudomonas* sp. ASC2	32.3 ± 0.3	61.8 ± 3.3	0.65	0.047	0.022	0.55
*Enterobacter* sp. ASC3	33.1 ± 0.2	47.2 ± 2.2	0.57	0.042	0.01	0.43
*Bacillus* sp. ASC4	13.2 ± 0.4	47.4 ± 1.5	0.51	0.022	0.017	0.17
TOC 20 g/L						
*Acinetobacter* sp. ASC1	27.3 ± 0.2	23.4 ± 2.7	0.31	0.023	0.014	0.18
*Pseudomonas* sp. ASC2	24.2 ± 0.1	39.4 ± 1.6	0.58	0.044	0.03	0.27
*Enterobacter* sp. ASC3	27.3 ± 0.2	30.0 ± 2.2	0.42	0.051	0.02	0.15
*Bacillus* sp. ASC4	21.8 ± 0.5	33.8 ± 2.4	0.41	0.023	0.023	0.2

Cells were grown in production medium with a carbon to nitrogen ratio of 200 in batch cultivation

**Fig. 4:**
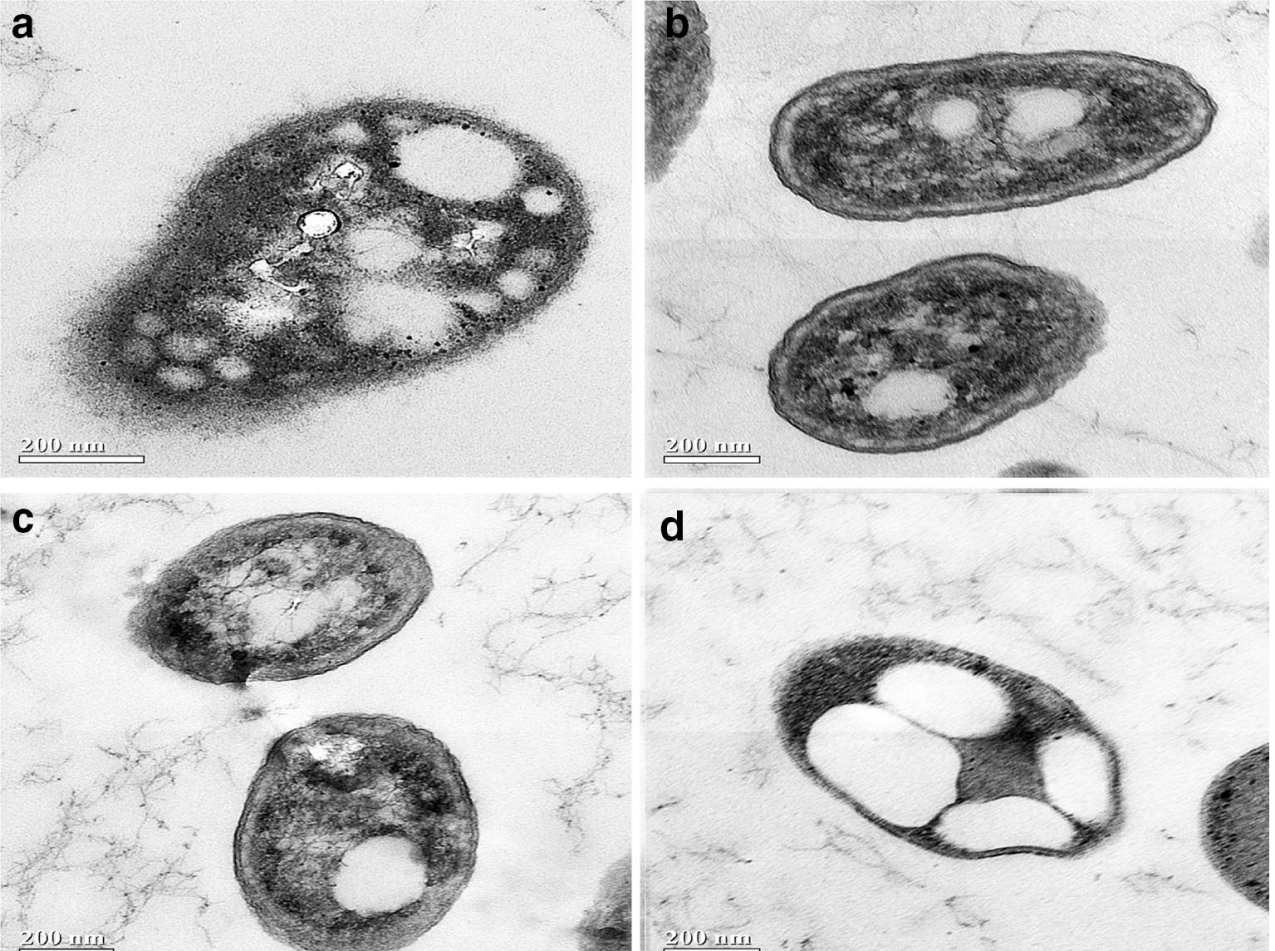
Transmission electron micrographs of an ultrathin section. **a** *Acinetobacter* sp. ASC1. **b** *Pseudomonas* sp. ASC2. **c** *Enterobacter* sp. ASC3. **d** *Bacillus* sp. ASC4. *Bars* 200 nm

Typical cell characteristics of *Acinetobacter* sp. ASC1, *Pseudomonas* sp. ASC2, *Enterobacter* sp. ASC3, *Bacillus* sp. ASC4, and the PHB granule content in cells, were examined by transmission electron microscopy (TEM). As can be seen in Fig. 4a, *Acinetobacter* sp. ASC1 cells were coccobacillus rods, 0.4 μm in diameter and 0.8–1.0 μm in length. PHA granules, numbering approximately 20, accumulated inside the cells. *Pseudomonas* sp. ASC2 cells were rods, 0.3 μm in diameter and 0.6–0.8 μm in length, containing a few PHA granules (Fig. 4b). TEM of *Enterobacter* sp. ASC3 cells (Fig. 4c) showed that they were short rods, 0.3 μm in diameter and 0.6 μm in length, containing 1–2 PHA granules. The TEM of *Bacillus* sp. ASC4 (Fig. 4d) revealed that the cells were short rods with 0.4 μm diameter and a length of 0.6 μm. PHA granules were clearly observed.

**Fig. 5:**
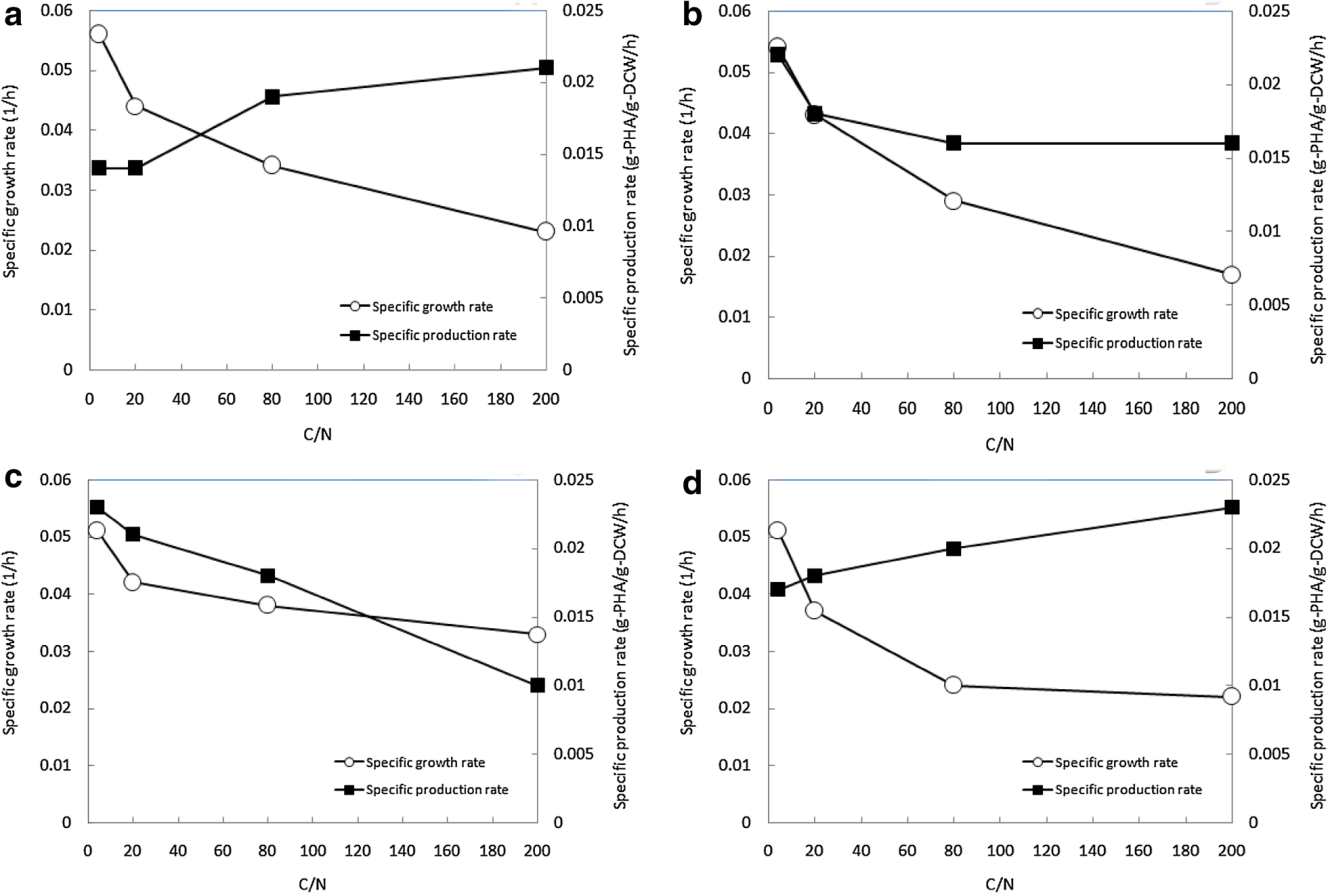
Effect of the C/N ratio on the specific growth rate of bacteria and specific production rate of PHA. **a** *Acinetobacter* sp. ASC1. **b** *Pseudomonas* sp. ASC2. **c** *Enterobacter* sp. ASC3. **d** *Bacillus* sp. ASC4

Next, a TOC concentration of 10 g/L was chosen, and the C/N ratio was varied (4, 20, 80, 100 and 200, respectively). Figure 5 shows the effect of the change in C/N ratio on specific growth rate, μ, and specific PHA production rate, ρ, for each bacterial strain. *Acinetobacter* sp. ASC1 (Fig. 5a) and *Bacillus* sp. ASC4 (Fig. 5d) attained their highest specific PHA production rates under nitrogen limited conditions (C/N = 200), whereas their specific growth rates reached their maximum under nitrogen sufficient conditions. In contrast, *Pseudomonas* sp. ASC2 (Fig. 5b) and *Enterobacter* sp. ASC3 (Fig. 5c) produced most PHA during their growth phase between a C/N of 4 and 20. These results correspond with the TEM analysis in Fig. 4. At a C/N ratio of 200, *Acinetobacter* sp. ASC1 (Fig. 4a) and *Bacillus* sp. ASC4 (Fig. 4d) accumulated many PHA granules, whereas *Pseudomonas* sp. ASC2 (Fig. 4b) and *Enterobacter* sp. ASC3 (Fig. 4c) harbored only a few PHA granules.

Based on GC analysis, the PHAs produced were not homopolymer PHB, but a copolymer of mcl-PHAs. Next, the mcl-PHAs were extracted from cells and subjected to chemical characterization.

### Characterization of mcl-PHAs by GC, ^1^H NMR, 2D-^1^H-COSY NMR and ^13^C NMR analyses

**Fig. 6:**
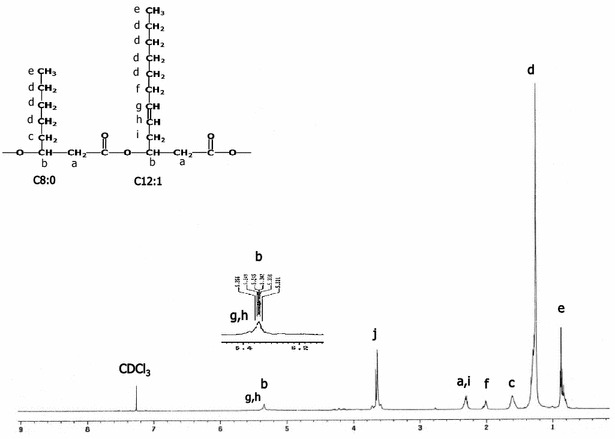
500 MHz ^1^H-NMR spectrum of P(5 % 3HO-co-95 % 3H5DD) synthesized by *Pseudomonas* sp. ASC2. Crude glycerol with a TOC concentration of 10 g/L and a C/N molar ratio of 200 were used in the culture. Letters on the spectrum indicate peaks arising from the protons marked in the corresponding structure. 3HO, 3-hydroxyoctanoate; 3H5DD, 3-hydroxy-5-cis-dodecanoate

**Fig. 7:**
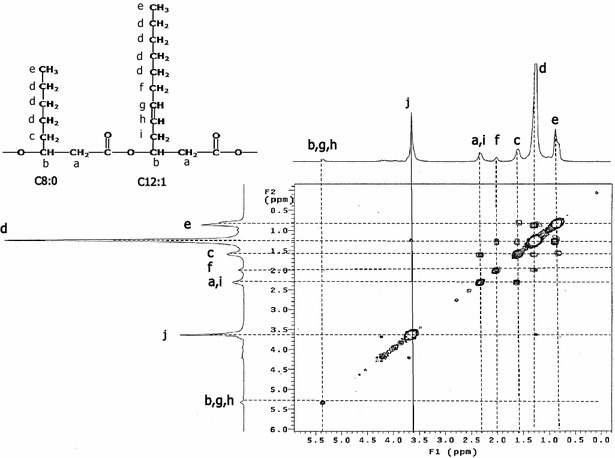
2D-COSY-^1^H NMR spectrum of P(5 % 3HO-co-95 % 3H5DD) synthesized by *Pseudomonas* sp. ASC2. Crude glycerol with a TOC concentration of 10 g/L and a C/N molar ratio of 200 were used in the culture. Letters on the spectrum indicate peaks arising from the protons marked in the corresponding structure. 3HO, 3-hydroxyoctanoate; 3H5DD, 3-hydroxy-5-cis-dodecanoate

To ensure an accurate determination of the PHA compositions, 5 L bioreactor batch cultivations containing 3 L production medium and an initial TOC concentration of 10 g/L with C/N ratio of 200 or C/N ratio of four depending on the bacterial strain were performed for each strain and the PHAs produced were purified as described in “Methods” section. GC was used to analyze the methyl esters of the monomers, and the 3-hydroxy fatty acid methyl esters were identified by comparison with various standards. Figures 6 and 7 show examples of ^1^H and 2D-^1^H-COSY spectra of mcl-PHAs, in this case those produced by *Pseudomonas* sp. ASC2 on batch cultivation in the bioreactor. NMR peak assignment was carried out according to previous literature [20–22]. The complex multiplet and unsymmetrical triplet observed respectively in regions *a* and *e* of the ^1^H spectrum (Fig. 6) are consistent with the overlap of peaks from monomer units found in all PHA samples. The ^1^H NMR spectra of all of the PHA samples produced in this work show peaks with almost identical chemical shifts that agreed well with those in previous reports [21, 22]. In Fig. 6, peaks *a* and *b* can be assigned to the protons next to the carboxyl group and the ether oxygen, respectively; peak *c* at 1.6 ppm was assigned to the first CH_2_ of the side chains; peaks *d* (at 1.3 ppm) and *e* (at 1.0 ppm) were assigned to remaining side-chain CH_2_ groups and the CH_3_ group, respectively. The chemical shifts of peaks *g* and *h* indicate the presence of an unsaturated group in some of the monomers. To identify peak *f* (at 2.0 ppm) and peak *j* (at 3.6 ppm), 2D-COSY-^1^H NMR spectroscopy was performed (Fig. 7). In 2D-COSY-^1^H NMR spectroscopy, cross peaks are present between coupled proton signals belonging to neighboring protons in the structure. A proton can walk through a chemical structure by proton to proton walking within the structure. In this way, the position of a double bound in an unsaturated alkyl chain can be identified. The peak *f* was assigned to a CH_2_ group next to a double bond [21]. Overall, this compound was identified as a copolymer consisting of 3-hydroxyoctanoate (3HO) and 3-hydroxy-5-*cis*-dodecenote (3H5DD) from peak *e* to peak *a* via peaks *b*, g and *h* in the ^1^H NMR and 2D-COSY-^1^H NMR spectra. Peak *j* was assumed to arise from impurities from other fatty acids because proton to proton walking through the structure could not be observed.

**Fig. 8:**
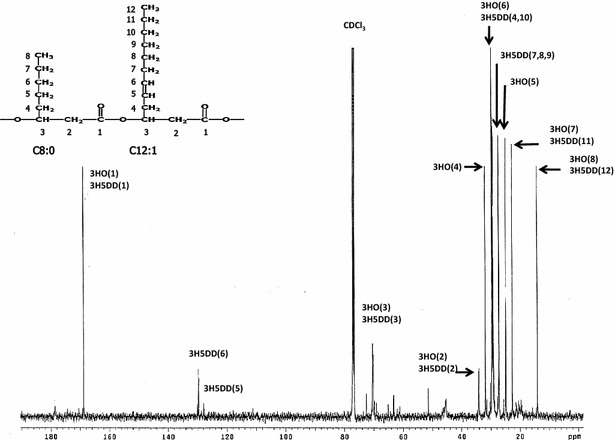
500 MHz ^13^C-NMR spectrum of P(5 % 3HO-co-95 % 3H5DD) synthesized by *Pseudomonas* sp. ASC2. Crude glycerol with a TOC concentration of 10 g/L and a C/N molar ratio of 200 were used in the culture. 3HO, 3-hydroxyoctanoate; 3H5DD, 3-hydroxy-5-cis-dodecanoate

**Fig. 9:**
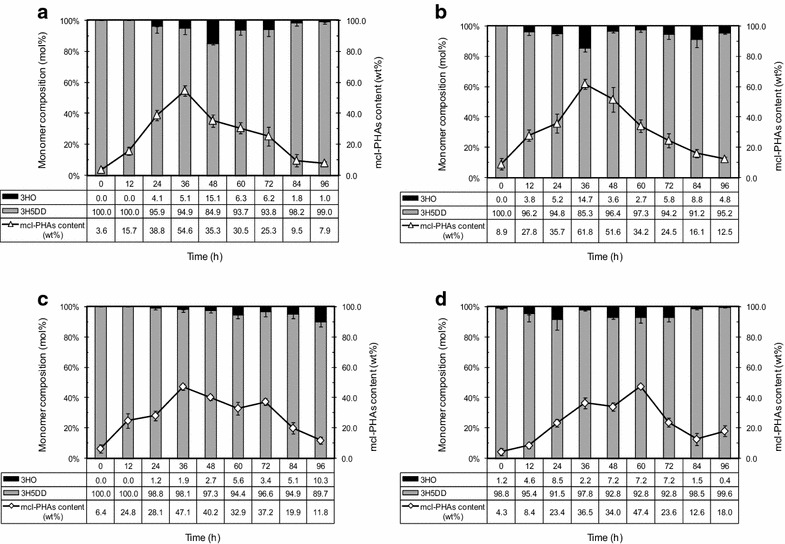
Time course profiles of mcl-PHA content (wt %) and monomer composition (3HO, 3-hydroxyoctanoate; 3H5DD, 3-hydroxy-5-*cis*-dodecanoate) (mol %). **a** mcl-PHA produced by *Acinetobacter* sp. ASC1. **b** *Pseudomonas* sp. ASC2. **c** *Enterobacter* sp. ASC3. **d** *Bacillus* sp. ASC4. Cells were grown on crude glycerol with a TOC concentration of 10 g/L and a C/N molar ratio of 200. Cultures were performed in triplicate. The *error bars* represent standard deviations

The PHA samples produced in this work were also studied by ^13^C NMR. Figure 8 shows the ^13^C NMR spectrum of poly(5 mol % 3-hydroxyoctanoate-co-95 mol % 3-hydroxy-5-*cis*-dedecanoate) [P(5 % 3HO-co-95 % 3H5DD)] synthesized by *Pseudomonas* sp. ASC2 from crude glycerol at a TOC concentration of 10 g/L and a C/N ratio of 200. The chemical shifts were assigned for each carbon resonance (see Table 5) according to previous reports [22, 23]. The main chain carbons (C_1_–C_3_, see Fig. 8) of the repeating units were assigned by comparison with the chemical shifts of carbons 1–3 of HO and 3H5DD and the values are given in Table 5 (previously reported in the literature [22, 23]). The chemical shifts at 14 and 31.9 ppm corresponded to the methyl groups of 3HO at positions C_4_ and C_5_ (see Table 5), respectively [23]. The chemical shifts at 129.9 and 128 ppm corresponded to the methyl groups of 3H5DD at positions C_6_ and C_5_, respectively [22]. The signals at 14 and 129.9 ppm confirmed the predominance of 3HO and 3H5DD units, thus matching the results of gas chromatography with flame ionization detection (GC–FID) analyses. The monomer composition profile, 3HO and 3H5DD, (mol %) analyzed by GC-FID is shown in Fig. 9. An increase of 3HO (mol %) composition with cultivation time was observed for *Enterobacter* sp. ASC3, whereas a decrease of 3HO (mol %) composition was observed for the other species. Homopolymeric 3H5DD was obtained from *Bacillus* sp. ASC4 when the cultivation time was prolonged to 96 h.

**Table 5:** Chemical shift data (in ppm) from ^13^C NMR spectra of PHA samples

Carbon^a^	Repeating units identified in PHA sample	
	3HO	3H5DD
1	169.4	169.4
2	34.1	34.1
3	70.8	70.9
4	32.1	30.2
5	27.8	128.1
6	31.5	130.1
7	22.5	28.2
8	14.0	28.3
9		28.4
10		30.5
11		22.5
12		14.0

^a^The number assignments for the carbons of the repeating units 3HO and 3H5DD are shown in Fig. 8

Huijberts et al. (1992) described the accumulation of products by *P. putida* KT2442 during growth on nonrelated substrates such as glucose, fructose and glycerol. In addition to the predominant monomer, 3HD, they observed that two of the monomers, 3-hydroxy-*cis*-5-dodecanoic acid (C_12:1_) and 3-hydroxy-*cis*-7-tetradecenoic acid (C_14:1_), contained unsaturated bonds [21]. Haywood et al. (1990) and Timm and Steinbuchel (1990) confirmed and extended this observation to show that the ability to accumulate PHA containing 3HD is shared by many *Pseudomonas* strains [20, 24]. In their studies, *P. mendocina* DSM 50017, the *Pseudomonas* species that presents the closest match to *Pseudomonas* sp. ASC2, could use gluconate as a carbon source and accumulated 50.7 wt % PHAs containing 3HHx (4.3 mol %), 3HO (29.8 mol %), 3HD (61.9 mol %) and 3HDD (4.2 mol %). More recently, several research groups have attempted to demonstrate metabolic pathways for *Escherichia coli* converting glucose [25] and *P. putida* converting relevant fatty acids [26] into mcl-PHAs composed primarily of 3-hydroxydodecanoate monomers.

In the present work, all mcl-PHAs were composed of two main monomers, 3HO (C_8:0_) and 3H5DD (C_12:1_), suggesting that the degradation pathway of medium chain fatty acid-containing substrates and the PHA synthesis pathway might be similar in all the tested strains. Based on the types of mcl-PHAs produced by the bacterial strains, we suggest that in the crude glycerol, which consists of glycerol 50.2 wt %, methanol 12.0 wt %, small amounts of saturated fatty acids [lauric acid (C_12:0_) 1.0 wt %, palmitic acid (C_16:0_) 5.4 wt %, stearic acid (C_18:0_) 1.6 wt %], and relatively high amounts of unsaturated fatty acids [oleic acid (C_18:1_) 11.9 wt %, linoleic acid (C_18:2_) 17.7 wt %, linolenic acid (C_18:3_) 1.8 wt %], the saturated fatty acids could be precursors for 3HO (C_8:0_), whereas the unsaturated fatty acids were the precursors for 3H5DD (C_12:1_). However, other medium length chain fatty acids such as 3HD-CoA (C_10:0_) were not observed in this study. It might be that the lack of 3HD-CoA (C_10:0_) was attributable to the β-oxidation of C_10_ that generates 3HO-CoA (C_8:0_) and also additional energy. In the presence of glycerol, the conversion of glycerol to acetyl-CoA may be used to generate either biomass or intermediates for the synthesis of mcl-PHAs, resulting in unsaturated medium chain fatty acid conversion to 3H5DD (C_12:1_) at a higher level than previously achieved. Based on GC-FID analysis combined with ^1^H NMR, 2D-COSY-^1^H NMR and ^13^C NMR spectra, we could not observe any monomers other than 3HO (C_8:0_) and 3H5DD (C_12:1_).

Various factors affect the production of mcl-PHA, including bacterial strains, types of carbon source, cultivation time, environmental conditions, PHA biosynthesis genes, and PHA biosynthesis pathways [27]. For *Bacillus* sp. and *Acinetobacter* sp., most reports are based on their ability to produce PHB from sugars and other unrelated carbon sources. Shahid et al. (2013) was the first report to disclose that *B. megaterium* DSM 509 possessed atypical metabolism resulting in mcl-PHA production from glycerol under conditions of nitrogen limitation. In this study, in specific cultivation conditions, mcl-PHA consisting of 3HB, 3-hydroxyhexanoate (3HHx), 3HO, 3HD, 3-hydroxydodecanoate (3HDD), 3H5DD, and 3-hydroxytetradecanoate (3HTD) was produced [28]. Hwan et al. (2008) also reported based on gas chromatography/mass spectroscopy analysis that *Acinetobacter* sp. strain DR1 could produce mcl-PHA consisting of 39 mol % 3HD, 52 mol % 3HDD and 9 mol % 3HTD. Although, there is no literature reporting the biosynthesis pathway for mcl-PHA production in these strains, we presume that our bacterial strains possess a *de novo* fatty acid biosynthesis pathway for the synthesis of PHA [29]. Huijberts et al. (1992) reported that the presence of both saturated and unsaturated hydroxyl fatty acids in PHA may result from a possible linkage between *de novo* fatty acid biosynthesis and PHA biosynthesis via β-oxidation [21]. Here, we report that *Acinetobacter* sp. ASC1 and *Bacillus* sp. ASC4 isolated in this study could produce mcl-PHAs with high 3H5DD content (>99 mol %) from crude glycerol. To our knowledge, there are a few reports regarding *Enterobacter* strains capable of PHA production [30–34]. Among them, *E. cloacae* SU-1 produced mcl-PHA consisting of 3HO and 3HHx using glucose or lactose [31], and *Enterobacter* strain FAK 1384 produced mcl-PHA containing 62 mol % 3HD, 18 mol % 3HO, 12 mol % 3H5DD, 7.6 mol % 3HDD, 0.3 mol % 3HHx and 1.3 mol % 3HTD using copra oil [34]. The present study is the first report that an *Enterobacter* strain accumulated PHAs using crude glycerol. *Enterobacter* sp. ASC3 accumulated 17.5 ± 0.2 g/L mcl-PHAs (total cell dry mass 33.1 ± 0.1 g/L with 47.2 ± 2.2 wt % PHA content) containing HO (10 ± 2.2 mol %) and 3H5DD (90 ± 2.7 mol %). The characterization of PHA biosynthesis genes in all these bacterial strains will be carried out in the future.

### Molecular mass distribution

The results of molecular mass measurements of the analyzed PHA samples, as estimated by gel permeation chromatography (GPC) experiments with polystyrene standards, are shown in Table 6. The highest values of number-average molecular weight (*M*_N_) and weight-average molecular weight (*M*_W_) were obtained from *Enterobacter* sp. ASC3 culture. The lowest *M*_N_ and *M*_W_ values were obtained from *Pseudomonas* sp. ASC2. The low molecular weight of the PHAs produced may be because waste glycerol causes the termination of chain propagation through the covalent esterification of glycerol to PHA in a chain terminating position [35, 36].

**Table 6:** Molecular weights of P(3HO-co-3H5DD) samples produced in batch cultivation

Sample	PHA composition (mol %)		Molecular weight		
	3HO	3H5DD	M_n_	M_w_	M_w_/M_n_
*Acinetobacter* sp. ASC1	1	99	4.7 × 10^4^	8.8 × 10^4^	1.9
*Pseudomonas* sp. ASC2	5	95	3.6 × 10^4^	6.5 × 10^4^	1.8
*Enterobacter* sp. ASC3	10	90	8.9 × 10^4^	14.5 × 10^4^	1.6
*Bacillus* sp. ASC4	0	100	6.6 × 10^4^	14.1 × 10^4^	2.2

*M*_*n*_ number average molecular weight, *M*_*w*_ weight average molecular weight, *M*_*w*_ */M*_*n*_ the polydispersity index, used as a measure of the broadness of a molecular weight distribution of a polymer. The larger the polydispersity index, the broader the molecular weight. Step polymerization reactions typically yield M_w_/M_n_ values of around 2.0

Notably, all of the mcl-PHAs produced in this study were amorphous polymers similar to the report by Song et al. (2008), which revealed that the major components 3HO, 3HD and 3HDD from waste vegetable oil are involved in the production of mcl-PHAs [37]. One possible factor in production of polymers with amorphous properties is the lack of PHB monomers incorporated in the copolymer and the use of saturated 3HO and unsaturated 3H5DD monomers. Polymer properties are highly dependent on factors such as the aliphatic chain lengths of the individual monomers, which in turn can impact crystallinity. A high number of amorphous regions may be induced by long chains and *cis*-type olefinic (C_12:1_) groups in the side chains, which both perturb the packing for crystallization. Therefore, the produced mcl-PHAs were not filmable polymers but were adhesive. Preusting et al. (1990) also reported that the presence of unsaturated rather than saturated end groups somehow prevented a crystalline arrangement of the polymeric chains, resulting in an amorphous polymer [38]. Interestingly, Pappalrado et al. (2014) applied a technique to prepare a film sheet by floating a toluene solution on a water surface and successfully obtained a transparent film of mcl-PHAs consisting of six monomers: 3HHx, 3HO, 3HD, 3HDD, 3H5DD and *cis* 3-hydroxydodec-6-enoate (3H6DD, C_12:1_Δ^6^) [17]. This technique may be applicable to the mcl-PHAs we produced. Sjögren et al. (2003) reported on the chemical characterization of four anti-fungal substances, 3-hydroxydecanoic acid, 3-hydroxy-5-*cis*-dodecenoic acid, 3-(R)-hydroxydodecanoic acid and 3-hydroxytetradecanoic acid, from *Lactobacillus plantarum* MiLAB 14. The mixtures of these 3-hydroxy fatty acids showed anti-fungal activities against different molds and yeasts with minimum inhibitory concentrations between 10 and 100 µg/mL [39]. Thus, mcl-PHAs consisting of a large percentage of unsaturated 3H5DD may be applicable in the food industry.

## Conclusions

The results presented herein permit the production of mcl-PHAs from waste glycerol by newly characterized bacterial strains screened from soil in Thailand. In this context, the interest in the production of mcl-PHAs lies in its potential use of waste glycerol which is otherwise a significant issue for waste water management. We highlight the high percentage of unsaturated monomers of 3H5DD obtained in this study, which may be useful for biomaterial applications in addition to serving in bioactive compounds to be used in the food industry.

## Methods

### Microorganisms

Bacteria were isolated from soil contaminated with used cooking oil in Thailand. The bacterial strains were maintained by monthly subculturing on nutrient agar slants. Stock cultures were maintained at −80 °C in a 15 % (v/v) glycerol solution.

### Carbon sources

Crude glycerol and refined glycerol were provided by Dr. Panu Punnarak, Witcorp Products Ltd. (a biodiesel factory at Tumbon Thasai, Amphur Muang, Samut-Sakorn, Thailand) during biodiesel manufacturing in 2010 and 2014. Used-cooking oil is used by this industry to produce biodiesel. The crude glycerol was used without any purification steps. The compositions of the crude glycerol and refined glycerol are shown in Table 2.

### Morphological and taxonomic identification

Cellular morphology after Gram-staining was checked using light microscopy. The Hucker method [40] was used for Gram-staining. Preliminary screening for PHA production was performed by comparative staining using modified Nile blue A [41, 42] and Sudan Black B [43] staining methods. Cell morphology and PHA granules were also observed by TEM. For TEM, diluted culture from production medium was fixed in 2 % (v/v) glutaraldehyde containing 2 % (v/v) paraformaldehyde in 0.1 M phosphate buffer (pH 7.2) and was postfixed in 1 % (w/v) osmium tetroxide. The cells were dehydrated in an ascending series of ethanol concentrations from 35 to 100 % and were embedded in Spurr resin (EMS, PA, USA). Thin sections were prepared with a LKB 2088 Ultratome V (Surrey, UK), stained with 2 % (w/v) uranyl acetate and 2 % (w/v) lead citrate and examined with a JEOL (TEM 2100) transmission electron microscope at an accelerating voltage of 80 kV. The catalase activity assay was performed by the detection of bubble formation in 3 % (w/v) hydrogen peroxide solution after incubating the cells in nutrient medium for 18–24 h. The ability to grow by utilizing substrates such as glucose, fructose, lactose, sucrose, maltose, and galactose was tested by inoculating the bacterial strains into basal medium supplemented with 2 % (w/v) of each carbohydrate, respectively; the cells were incubated at 30 °C for 3 days. Starch hydrolysis, gelatin hydrolysis, hydrogen sulfide production, nitrate reduction, the Methyl Red-Voges Proskauer (MR-VP) test, and the citrate utilization test were performed. Acid production from carbohydrates was determined in basal medium that was supplemented with various carbohydrates [44]. All assays were performed three times.

## *16S rDNA* gene sequencing

Genomic DNA was prepared from colonies using the Wizard Genomic DNA Purification Kit (Promega, USA) and was used for PCR. 16S rDNA was amplified using the primers 27F (5′-AGAGTTTGATCMTGGCTCAG-3′) and 1492R (5′-TACGGYTACCTTGTTACGACTT-3′). The PCR reaction and 16S rDNA sequencing were performed at the Macrogen service center (Macrogen Inc., Seoul, Korea). To avoid misreading due to PCR error, sequencing of the PCR fragments was repeated at least twice. The BLASTN program (http://www.ncbi.nlm.nih.gov/BLAST/; NCBI, Bethesda, MD, USA) was used for gene homology searching with the standard default parameters.

### Phylogenetic analysis of *16S rDNA* gene sequences

The nucleotide sequences obtained were analyzed using DNASIS-MAC software (version 2.05; Hitachi Software Engineering Co. Ltd., Yokohama, Japan). Multiple alignments of the determined sequences were performed using the CLUSTAL X program, version 1.83 [45]. Phylogenetic trees were constructed by the neighbor-joining method [46], and phylogenetic analysis was performed using the PHYLIP version 3.572c package [47]. The percentage confidence was estimated by bootstrap analysis with 100 replications.

### Culture conditions

Shaken flask experiments were performed in 250-mL Erlenmeyer flasks containing 50 mL of medium. The preculture medium was the same as previously described [48]. The pre cultures were grown on a rotary shaker (200 rpm) at 30 °C for 24 h. Cells were harvested by centrifugation, washed to remove the nitrogen source and resuspended in 100 mL of 0.85 % sodium chloride solution. The cells were separately inoculated into production medium, a mineral salt medium [48] containing total organic carbon at varying concentrations (5, 10 or 20 g/L). The cultivation was performed in 500-mL Erlenmeyer flasks containing 100 mL of production medium with shaking at 200 rpm at 30 °C for 96 h, and culture samples were taken at 12 h intervals. The effect of the mole ratio of C/N on the relationship between specific growth rate, μ (1/h), and specific production rate, ρ (g PHA/g CDM/h), was investigated in detail for C/N values of 4 and 200 and without a nitrogen source.

For batch experiments, 500 mL of seed culture were prepared in flasks and grown on a rotary shaker at 30 °C at 200 rpm for 24 h. The cells were harvested by centrifugation, washed to remove the nitrogen source and resuspended in 100 mL of 0.85 % NaCl solution. The cells were then inoculated into a synthetic medium containing 3.4 g/L KH_2_PO_4_, 5.8 g/L K_2_HPO_4_, 0.12 g/L MgSO_4_·7H_2_O, 5.0 g/L Na_3_C_6_H_5_O_7_, and 1 mL of trace elements solution [1.67 g/L CaCl_2_·2H_2_O, 2.78 g/l ZnSO_4_·7H_2_O, 0.29 g/L FeSO_4_·7 H_2_O, 1.98 g/L MnCl_2_·4H_2_O, 0.17 g/L CuCl_2_·2H_2_O] in a 5-L bioreactor (MBF-500ME, EYELA, Tokyo Rikakikai Co. Ltd., Tokyo, Japan) that was interfaced with an EPC control box (EPC-1000, EYELA, Tokyo Rikakikai Co. Ltd.). The working volume of the batch cultures was 3 L. The fermentation temperature was 30 °C, and the pH value was maintained at 7.0 throughout the experiments. The values for these parameters were monitored and recorded using the online program TK97 Data Record version 2.04 (EYELA, Tokyo Rikakikai Co. Ltd.).

### Analytical methods

Growth was calculated using cell dry mass. Culture broth was centrifuged at 3000×*g* for 10 min. The supernatant was used to determine total organic carbon concentrations. The cell pellet was washed with acetone and centrifuged at 3000×*g* for 10 min to remove glycerol and fatty acid residue. The cells were suspended in 0.85 % NaCl solution and the cell suspension was filtered through pre weighed cellulose nitrate membrane filters (pore size 0.22 μm, Sartorius, Goettingen, Germany) and dried at 80 °C for 2 days. The net biomass was defined as the residual biomass, which was calculated by subtracting the amount of PHA from the total biomass. The PHAs in dried cells were methyl-esterified in a mixture of chloroform and 3 % methanol-sulfuric acid (1:1 v/v), as described by Braunegg [49]. The resulting methyl esters of the monomers were quantified using GC (Model CP3800, Varian Inc., Walnut Creek, CA, USA) with a Cabowax-PEG capillary column (0.25 μm df, 0.25 mm ID, 60 m length, Varian Inc.). The internal standard was benzoic acid, and the external standards were PHB, PHBV, n-octanoic acid and n-decanoic acid (Sigma-Aldrich Corp., St. Louis, MO, USA). The PHA compositions and the mole fractions were confirmed by ^1^H and ^13^C NMR (Varian Inova 600 MHz; Palo Alto, CA, USA). The NH_4_^+^ concentration in the culture medium was determined using a colorimetric assay [50]. To determine the quantities of total carbon (TC), inorganic carbon (IC) and organic carbon (TOC), crude glycerol was analyzed with a Total Organic Carbon Analyzer (Shimadzu TOC-Vcsh, Shimadzu Corporation, Nakagyo-ku, Kyoto, Japan) at the Petroleum and Petrochemical College of Chulalongkorn University, Bangkok, Thailand. The total carbon in the crude glycerol was 300.9 g/L.

### PHA extraction and purification

Harvested cells were dried and packed in filter paper (Whatman 1002–042) and refluxed in hot chloroform in a Soxhlet to extract the PHAs from the dried cells. The PHAs were recovered from the chloroform by precipitation in *n*-hexane. The precipitation step was repeated three times [51].

### Chemical structure analysis

^1^H and ^13^C NMR spectra of PHA samples were recorded on a Varian Inova 500 MHz instrument. The chemical shifts are reported in parts per million (ppm) relative to chloroform as an internal reference. Spectra were recorded using 5 % (w/w) polymer solutions in CDCl_3_ with the following parameters: 25 °C, pulse of 90°, width of 8003.2 Hz, 5.0 s relaxation delay, and 0.2 Hz line broadening. Two dimensional-^1^H-correlation spectroscopy (2D-^1^H-COSY) was performed in combination with ^1^NMR on a Bruker AM 400 MHz FT-NMR (BrukerBioSpin Corporation, Woodland, TX, USA).

### Measurement of the molecular weight distribution of PHAs

The molecular weight of PHAs was determined by a GPC system with a refractive index detector (Class-VP series with software version V6.14; Shimadzu Corp., Tokyo, Japan) using a Styragel HT6E column (Waters, Milford, MA, USA). Chloroform was used as the elution solvent at a flow rate of 1.0 mL/min. The operating temperature was maintained at 40 °C. The calibration curve was determined using low polydispersity polystyrene standards (*M*_W_ = 4.56 × 10^2^, 2.98 × 10^3^, 3.79 × 10^4^, 9.64 × 10^4^, 1.90 × 10^5^, and 7.06 × 10^5^; Tosoh Corp., Tokyo, Japan). The GPC data were calculated using an integrator for the number average molecular weight (*M*_N_), the weight average molecular weight (*M*_W_), and the polydispersity index (*M*_W_/*M*_N_).

## Abbreviations

C/N: molar ratio of carbon to nitrogen; PHA: polyhydroxyalkanoate; CDM: cell dry mass;RCM: residual-cell mass; 3HB: 3-hydroxybutyrate (C_4_); 3HHx: 3-hydroxyhexanoate (C_6_); 3HO: 3-hydroxyoctanoate (C_8_); 3HD: 3-hydroxydecanoate (C_10_); 3HDD: 3-hydroxydodecanoate (C_12:0_); 3H5DD: 3-hydroxy-5-*cis*-dodecanoate (C_12:1_Δ^5^); 3H6DD: 3-hydroxy-6-*cis*-dodecanoate (C_12:1_Δ^6^); 3HTD: 3-hydroxytetradecanoate (C_14_).

### Greek letters

*Μ*: specific growth rate (1/h); *Ρ*: specific production rate of polymer (g PHA/g CDM/h).
